# Assessment of HTLV-I proviral load, HIV viral load and CD4 T cell count in infected subjects; with an emphasis on viral replication in co-infection

**Published:** 2014-01

**Authors:** Hossein Rahimi, Seyyed Abdolrahim Rezaee, Narges Valizade, Rosita Vakili, Houshang Rafatpanah

**Affiliations:** 1 Hematology Department, Ghaem Hospital, Faculty of Medicine, Mashhad University of Medical Sciences, Mashhad, Iran; 2 Immunology Research Center, Faculty of Medicine, Mashhad University of Medical Sciences, Mashhad, Iran; 3 Inflammation and Inflammatory Diseases Research Center, Faculty of Medicine, Mashhad University of Medical Sciences, Mashhad, Iran

**Keywords:** HIV viral load, HTLV-I/HIV co-infection, HTLV-I proviral load, Lymphocytes

## Abstract

***Objective(s):*** HTLV-I and HIV virus quantification is an important marker for assessment of virus activities. Since there is a direct relationship between the number of virus and disease progression, HTLV-I and HIV co-infection might have an influence on the development of viral associated diseases, thus, viral replication of these viruses and co-infection were evaluated.

***Materials and Methods:*** In this study, 40 subjects were selected; 14 HIV infected, 20 HTLV-I infected and 6 HTLV-I/HIV co-infected subjects. The amount of viruses was measured using qPCR TaqMan method and CD4 and CD8 lymphocytes were assessed by flow cytometry.

***Results:*** The mean viral load of HIV infected subjects and HTLV-I infected individuals were 134626.07±60031.07 copies/ml and 373.6±143.3 copies/10^4^ cells, respectively. The mean HIV viral load in co-infected group was 158947±78203.59 copies/ml which is higher than HIV infected group. The mean proviral load of HTLV-I in co-infected group was 222.33±82.56 copies/ml which is lower than HTLV-I infected group (P<0.05). Also, the mean white blood cell count was higher in co-infected group (5666.67±1146.49 cells/μl). However, the differences between these subjects did not reach to a statistical significance within 95% confidence interval level (*P* =0.1). No significant differences were observed regarding CD4 and CD8 positive lymphocytes between these groups.

***Conclusion:*** HTLV-I/HIV co-infection might promote HIV replication and could reduce the HTLV-I proviral load, in infected cells. Considering the presence of both viruses in Khorasan provinces, it encourages researchers and health administrators to have a better understanding of co-infection outcome.

## Introduction

Human immunodeficiency virus (HIV) is a lentivirus belonging to *Retroviridae* family. This virus mostly infects CD4+ T lymphocytes which have a crucial role in immune regulation and causes immunodeficiency syndrome (AIDS) by severe lymphocyte depletion. A global total of 40 million people were diagnosed with HIV infection in 2012 and almost 5 million people were reported as newly infected individuals (-). Based on the AIDS progress report in 2012, HIV prevalence in Iran has been significantly increased. Therefore, effective control and prevention of HIV and also appropriate monitoring of HIV infected individuals must be implemented as major health programs in Iran (4). Since plasma HIV-I RNA level is an independent predictor of HIV disease progression, its measure-ment is important to evaluate the efficacy of antiretroviral drug therapies and to monitor disease progression in HIV infected individuals (5-6).

Human T lymphotropic virus type I (HTLV-I) is a retrovirus belonging to *Retroviridae* family and associated with two main types of diseases; adult T-cell leukemia (ATL) and the inflammatory condition named HTLV-I-associated myelopathy/tropical spastic paraparesis (HAM/TSP) (7-8). This virus is endemic in several regions of the world, such as southwest of Japan, the Caribbean basin, Central Africa, South America, the Melanesian Islands and the Middle East (9-10). HTLV-I is endemic in at least 5 provinces of Iran including Khorasan Razavi, Northern Khorasan, Golestan, Alborz and Eastern Azarbayejan (-). The prevalence of HTLV-I infection in Iran is estimated to vary among 24 provinces (14).

HTLV-I proviral load is a major determinant of HTLV-I infection outcome and higher HTLV-I proviral load as well as the interaction between the virus and the host have crucial role in the development of HTLV-I associated diseases (15-16).

Both HTLV-I and HTLV-II are frequent co pathogens among individuals infected with HIV-I in different parts of the world. HTLV-I and HIV share similar routes of transmission and predominantly are CD4+ T-cell tropic (8, -). Several studies have demonstrated a protective effect on AIDS progression by HTLV-II (8, 17, 20). *Furthermore*, an increased frequency of other clinical complications has been reported in HIV–HTLV-I/II co-infection (21). Some laboratory evidences suggested that AIDS progression is promoted by HTLV-I/HIV-I co-infection. However, the net impact of HTLV-I/HIV-I co-infection on HIV disease is *still controversial (*8*, *22*).* It seems that introduction of highly active antiretroviral therapy (HAART), has been led to longer survival for HIV-infected individuals and stops HIV replication, but does not have any protective impact on HTLV proviral load, CD4 cell count and HAM/TSP development (18-19, 23-25). 

The *obvious* outcome of HTLV-I or HTLV-II and HIV co-infection is an increased CD4^+^ cells count without any immune benefit for patients (26-27). Furthermore, some studies have reported that the risk of developing HAM/TSP in HIV–HTLV-I co-infected patients is higher than in HTLV-I-infected individuals and it may be due to the higher HTLV proviral load in co-infected patients (18, 22, 28-29). Understanding the effect of HTLV-I virus on HIV viral replication and vice versa, will help for better monitoring and following up of HIV and HTLV-I–associated diseases in this endemic area. 

## Materials and Methods

***Study design***

A total of 112 HIV positive subjects were recruited from triangular clinic, Mashhad University of Medical Sciences, Mashhad, Iran from January 2001 to 2010. This clinic is the only one in Khorasan Razavi province providing a wide-range of counseling and treatment for HIV positive subjects. Only the patients who were asymptomatic at the time of admission to the clinic (WHO stage I) and those newly diagnosed asymptomatic HTLV-I cases were included in this study. Including criteria were as follow; newly diagnosed untreated subjects, non IV drug users, Khorasani residents and healthy carriers without any diseases symptoms at the time of sampling. Forty subjects met our study criteria including; 14 HIV, 20 HTLV-I and 6 HTLV-I/HIV co-infected subjects. For each subject, clinical staging was assigned based on the revised criteria of WHO staging system (30) by an infectious disease specialist.

The study was approved by the Research Ethics Committee of Mashhad University of Medical Sciences (No: 88797). Participants had full authority to participate in study. An informed consent was taken from participants. All information were recorded and tagged with proper identification codes to identify participants for confidentiality. 

Specialized physicians examined the patients and recorded the results that were including complications related to immune system (infections, malignancies*, lymphadenopathy, ext.)* and general complications. All participants were completed a standard questionnaire including demographic information and the history of any current illness. 

***Sample collection and serological assay***

Blood sample of 10 ml was collected from each participant. Serum samples were tested for the presence of anti-HTLV-I (Diapro, Italy) and anti-HIV (Diapro, Italy) by enzyme-linked immunosorbent assay (ELISA) according to manufacturer instruct-tions in Center of Infectious Diseases (Mashhad, Iran). Positive specimens were confirmed by a conventional polymerase chain reaction (PCR). To confirm HTLV-I infection a conventional PCR was carried out for Tax and LTR region on PBMCs extracted DNA. Genomic DNA was extracted from peripheral blood mononuclear cells (PBMCs) using an available commercial kit (Blood mini kit, Qiagen, Germany) and PCR amplification was performed using specific primers for tax (5-AGGGTTTGGACA GAGTCTT-3 and 5-AAGGACCTTGAGGGTCTTA- 3) and LTR regions (5-CATAAGCTCAGACCTCCGGG-3 and 5- GGATGGCGGCCTCAGGTAGG-3).

***HTLV-I proviral load***

To assess the HTLV-I proviral load, PBMCs were isolated from EDTA-treated blood samples by Ficoll density gradient (Sigma, Germany). Genomic DNA was extracted from PBMCs using an enzymatic method (QIAamp® DNA Mini Kit) according to the manufacturer instructions. A real time PCR (Taqman probe) using an absolute quantification kit was carried out to measure the HTLV-I proviral load in PBMCs using specific primers and a fluorogenic probe which have been tested on Rotorgen Q 6000 machine (Qiagen, Germany) The HTLV-I copy number was reported as an actual amount of cellular DNA by means of the quantification of the albumin gene as the reference gene. HTLV-I and albumin DNA concentrations were calculated from two 5-point standard curves. The normalized value of the HTLV-I proviral load was calculated as the ratio of (HTLV-I DNA copies number/albumin DNA copies number/2) ×10^4^ and expressed as the number of HTLV-I provirus per 10^4^ PBMCs (31).

***HIV viral load***

To examine the HIV viral load, RNA was extracted from plasma using Viral RNA extraction Mini Kit (Qiagen, Germany). A Real Time PCR (Taqman probe) using a commercial absolute quantification kit (FTD Kit, Luxembourg) was carried out to measure HIV viral load by a Rotorgen Q machine (Qiagen, Germany). 

***Evaluation of CD4 and CD8 counts***

CD4, CD8 and CD3 cells were stained by conjugated antibodies FITC, PE and PreCP, respectively (IQ products, the Netherlands). Cells were analyzed using a FACS Calibour (BD Bioscience, USA) flow cytometer.

***Statistical analysis***

Data were analyzed by Mann-Whitney and Independent Sample T Tests using SPSS/ver.16 software. Descriptive data were summarized as mean with standard deviation (SD) and standard error of mean (SEM). *P*- values of < 0.05 were considered statistically significant.

## Results

Thirty seven subjects with mean age of 35.88 ± 1.89 years (ranging 22-50 years) were studied. The mean age of HIV, HTLV-I and HTLV-I/HIV co-infected subjects were 36.43 ± 2.66, 35.71 ± 3.87 and 33.67 ± 2.33 years, respectively.

The mean HTLV-I proviral load in HTLV-I infected individuals was 373.6 ± 143.3 and in HTLV-I/HIV co-infected group was 222.333 ± 82.56, the difference was statistically significant (*p* < 0.05). The mean HIV viral load in HIV group was 134626.07 ± 60031.07 and in HTLV-I/HIV co-infected group was 158947 ± 78203.59. There was no significant difference between two groups.

Table 1 shows the mean HTLV-I proviral load, mean HIV viral load, and mean age of patients in HTLV-I- infected group, HIV infected subjects and HTLV-I/HIV co-infected groups. 

The results of this study show that HIV viral load in HTLV-I/HIV co-infected patients (158947 ± 78203.59) was higher than in HIV infected patients (134626.07 ± 60031.07). Furthermore, HTLV-I proviral load in HTLV-I/HIV co-infected patients (222.3333 ± 82.56) is lower than in HTLV-I infected patients (373.6 ± 143.3) (*p* < 0.05). 

The mean white blood cell count was higher in co-infected group (5666.67±1146.492 cells/μL) than both groups (P = 0.1). However, the differences between these subjects did not reach a statistical significance within 95% confidence interval (*p* = 0.1) level.

The CD4+ cells count in HTLV-I infected (659.9 ± 110.7) and co-infected patients (431.43 ± 120) was higher than HIV infected group (414 ± 97.5), however, the differences were not statistically significant.

## Discussion

HIV and HTLV-I are two human retroviruses that mostly contaminate CD4+ helper lymphocytes. HTLV-I/HIV co-infection has been reported in different parts of the world. Since there is a direct relationship between HIV- RNA plasma level and the clinical progression of AIDS. High HTLV-I proviral has been detected in HTLV-I related diseases which are implicated in clinical progression and severity of these diseases (8, 22, 32-35). 

Table 1 shows HIV viral load in HTLV-I/HIV co-infected patients is higher than in HIV infected patients. Some studies revealed that HTLV-I is associated with HIV progression and shorter survival time (36-37). Tax protein in HTLVI virus is responsible for cell cycle regulation and viral transcription. Furthermore, it has been shown that Tax protein upregulates HIV replication and expression of cytokines and their receptors which attribute to T-cell activation and finally leads to the progression of HIV-I infection in co-infected patients (38). In a case control study conducted in Brazil, HIV viral load was higher in HTLV-I/HIV co-infected group as compared to HIV group and it was positively associated with an enhancement of T-cell activation markers in both co-infected and HIV groups (37). These findings have also been confirmed by previous studies (21, 39-40). However, Harrison et al reported HTLV-I has no effect on HIV viral load. In this study, HIV viral load was higher in HIV infected group than HTLV-I/HIV co-infected group. This inconsistency might be due to the incidence of different HIV-I phenotypes in clinical disease (38, 41). Since soluble factors secreted by HTLV-I infected cells determine progression or prevention of HIV-I infection, the real impact of HTLV-I/HIV co-infection on HIV pathogenesis is still controversial (8, 38). 

**Table1:** Mean HTLV-I proviral load, mean HIV viral load and mean age in HTLV-I infected patients, HIV infected and HTLV-I/HIV co-infected groups

Variables	Gender		
	Total (n=24)	Female (n=7)	Male (n=17)
HIV infected group	5214.29±495.680	3950±28.86	5720±630.30
HTLV-I infected group	5657.14±374.711	5933.33±788.10	5450±384.05
HIV /HTLV-I co-infected group	5666.67±1146.49	_________	5666.67±1146.49
HTLV-I proviral load (mean ± SE) Copy number/10^4^ PBMC			
HTLV-I infected group	373.6±143.3	485±300.88	289.75±75.06
HIV /HTLV-I co-infected group	222.33±82.56	_________	222.33±82.56
HIV viral load (mean ± SE) copies/mL of plasma			
HIV infected group	134626.07±60031.07	83591.25±48007.03	155040±82572.33
HIV /HTLV-I co-infected group	158947±78203.59	_________	158947±78203.59
Age (mean years)			
HIV infected group	36.43±2.66	30.75±3.61	38.70±3.25
HTLV-I infected group	35.71±3.87	30±2.88	40±5.83
HIV /HTLV-I co-infected group	33.67±2.33	___________	33.67±2.33

WBC count: **white blood cell count**, SEM: standard error of mean, PBMCs: peripheral blood mononuclear cells

In the present study, HTLV-I proviral load in HTLV-I infected patients was higher than co-infected patients (Table1), which was statistically meaningful. Similar results were obtained by Cesaire (42). It has been demonstrated that HIV may activate HTLV-I viral expression by interaction with host cellular genes in co-infected patients and as a result could accelerate HAM/TSP development in infected individuals (18, 43-44), however, the results of our study did not show such effects. 

Helper T lymphocytes (CD4+) are the main targets for HTLV-I and HIV and in case of HTLV-I and HIV; it leads to T cell proliferation (7, 8) and depletion, respectively (1). Therefore, it was assumed that the CD4 T cell count would be higher in HTLV-I infected groups. In our study, CD4+ T cells count in HTLV-I infected and co-infected groups was higher than HIV group and this difference was not statistically meaningful, although previous studies have reported that CD4+ T cell count in HTLV-I/HIV co-infected patients is higher than HIV infected individuals (19, 26, 29, 45). 

In a recent study, evaluation of absolute lymphocyte count among HTLV-I/HIV co-infected and HIV infected groups showed that, lymphocyte reduction in co-infected patients occurs more slowly than in HIV infected group. This could explain the higher HTLV-I proviral load in co-infected patients due to increasing number of host cells for HIV virus. In contrast, progressive depletion of CD4+ T cells (target cells for HTLV-I virus infection) might explain the reduction of HTLV-I provirus load in the co-infected patients (37). 

Furthermore, in the present study like some previous results, HTLV-I proviral load in women was higher than men and in contrast, HIV viral load in men was higher than women (Table 1) which may be due to the effects of male hormones on immune system, particularly lymphocyte count (31, 46-50). Since, in the present study, there have been some confounding factors such as nutrition, genetic variations and economic situation, the authors tried to minimize these factors by choosing suitable control groups, HIV positive subjects in stage one, newly diagnosed cases of HIV or HTLV-I. Further studies with larger sample size might help to clarify the role of viral and host factors in HIV/HTLV-I co-infections. 

## Conclusion

HTLV-I/HIV co-infection could possibly increase the HIV viral load and reduce the HTLV-I proviral load. Since HTLV-I/HIV co-infection is increasing in different parts of the world, larger studies should be conducted to discover the interactions between HIV and HTLV-I and host to have a better understanding of clinical co-infection outcome, transmission and monitoring HTLV-I/HIV co-infection subjects.
